# Thrombotic Events in COVID-19 Are Associated With a Lower Use of Prophylactic Anticoagulation Before Hospitalization and Followed by Decreases in Platelet Reactivity

**DOI:** 10.3389/fmed.2021.650129

**Published:** 2021-04-22

**Authors:** Chantal C. Clark, Bernard N. Jukema, Arjan D. Barendrecht, Judith S. Spanjaard, Nikita K. N. Jorritsma, Simone Smits, Steven de Maat, Cor W. Seinen, Sandra Verhoef, Naomi M. J. Parr, Silvie A. E. Sebastian, Arnold C. Koekman, Annet C. W. van Wesel, Harriet M. R. van Goor, Roy Spijkerman, Suzanne H. Bongers, Erhard van der Vries, Stefan Nierkens, Marianne Boes, Leo Koenderman, Karin A. H. Kaasjager, Coen Maas

**Affiliations:** ^1^Department of Central Diagnostic Laboratory Research, University Medical Center Utrecht, Utrecht University, Utrecht, Netherlands; ^2^Center for Translational Immunology, University Medical Center Utrecht, Utrecht University, Utrecht, Netherlands; ^3^Department of Internal Medicine, University Medical Center Utrecht, Utrecht University, Utrecht, Netherlands; ^4^Department of Respiratory Medicine, University Medical Center Utrecht, Utrecht University, Utrecht, Netherlands; ^5^Department of Trauma Surgery, University Medical Center Utrecht, Utrecht University, Utrecht, Netherlands

**Keywords:** COVID-19, platelets, SARS-CoV-2, thrombosis, thrombotic event, pulmonary thrombosis, thromboinflammation

## Abstract

**Background:** Coronavirus disease of 2019 (COVID-19) is associated with a prothrombotic state and a high incidence of thrombotic event(s) (TE).

**Objectives:** To study platelet reactivity in hospitalized COVID-19 patients and determine a possible association with the clinical outcomes thrombosis and all-cause mortality.

**Methods:** Seventy nine hospitalized COVID-19 patients were enrolled in this retrospective cohort study and provided blood samples in which platelet reactivity in response to stimulation with ADP and TRAP-6 was determined using flow cytometry. Clinical outcomes included thrombotic events, and all-cause mortality.

**Results:** The incidence of TE in this study was 28% and all-cause mortality 16%. Patients that developed a TE were younger than patients that did not develop a TE [median age of 55 vs. 70 years; adjusted odds ratio (AOR) = 0.96 per 1 year of age, 95% confidence interval (CI) 0.92–1.00; *p* = 0.041]. Furthermore, patients using preexisting thromboprophylaxis were less likely to develop a thrombotic complication than patients that were not (18 vs. 54%; AOR = 0.19, 95% CI 0.04–0.84; *p* = 0.029). Conversely, having asthma strongly increased the risk on TE development (AOR = 6.2, 95% CI 1.15–33.7; *p* = 0.034). No significant differences in baseline P-selectin expression or platelet reactivity were observed between the COVID-19 positive patients (*n* = 79) and COVID-19 negative hospitalized control patients (*n* = 21), nor between COVID-19 positive survivors or non-survivors. However, patients showed decreased platelet reactivity in response to TRAP-6 following TE development.

**Conclusion:** We observed an association between the use of preexisting thromboprophylaxis and a decreased risk of TE during COVID-19. This suggests that these therapies are beneficial for coping with COVID-19 associated hypercoagulability. This highlights the importance of patient therapy adherence. We observed lowered platelet reactivity after the development of TE, which might be attributed to platelet desensitization during thromboinflammation.

## Introduction

COVID-19 (coronavirus disease 2019) is associated with pulmonary symptoms, including pneumonia or Acute Respiratory Distress Syndrome (ARDS) (1). The development of severe disease correlates with hemostatic abnormalities such as a mild thrombocytopenia and increased D-dimer concentrations (2–4). This is accompanied by increased concentrations of fibrinogen, factor VIII, and von Willebrand factor, as well as fibrinolytic shutdown have been reported in hospitalized COVID-19 patients (5). Normal thrombin generation despite prophylactic heparinization of patients is another example of the hypercoagulability that takes place during SARS-CoV-2 infection. This helps to explain the frequently occurring thrombotic events (TE) in hospitalized COVID-19 patients, despite thromboprophylaxis (6–8). The most common observed thrombotic event is a pulmonary embolism (PE) (6) which can occur in the absence of deep vein thrombosis (DVT) (7, 8), potentially referring to *in situ* pulmonary thrombosis. Furthermore, platelet hyperreactivity might also contribute to the development of TE, as increased baseline platelet activation markers, and increased platelet reactivity have been reported in these patients (9–12). Most of these studies investigated disease severity as main clinical outcome, rather than TE, and compared healthy volunteers with COVID-19 patients. Here, we explored whether changes in platelet reactivity are associated with TE risk or all-cause mortality in hospitalized COVID-19 patients.

## Methods

### Reagents

Adenosine diphosphate (ADP) was from Sigma-Aldrich (Zwijndrecht, the Netherlands). Allophycocyanin (APC)-conjugated monoclonal Mouse Anti-Human P-selectin (CD62P) antibody clone AK4, Phycoerythrin (PE) conjugated monoclonal Mouse Anti-Human P-selectin antibody clone AK4, BD FACSCanto II, and FACSCanto II Diva software version 8.0.1 were from BD Biosciences (Franklin Lakes, New Jersey, USA). Fluorescein isothiocyanate (FITC) conjugated polyclonal Rabbit Anti-Human fibrinogen antibody (F011102-2) was from Dako (now Agilent, Santa Clara, CA, USA).

Formaldehyde (37%) was from Calbiochem (San Diego, California, USA). MgSO4 was from Merck (Darmstadt, Germany). NaCl, KCl were from Sigma–Aldrich (St. Louis, MO, USA). PAR (protease-activated receptor)-1 agonist SFLLRN (TRAP-6) was from Bachem (Bubenhof, Zwitserland). 1.2-mL polypropylene tubes were obtained from BRAND GmbH & Co. KG (Wertheim, Germany). 4-(2-hydroxyethyl)-1-piperazineethanesulfonic acid (HEPES) was from VWR International (Amsterdam, The Netherlands). Ninety-six-well PS flat-bottom plates were from Greiner Bio-one (Alphen aan den Rijn, The Netherlands).

### Study Design

Hospitalized patients (≥18 years old) admitted to the University Medical Center Utrecht between March 17th and May 1st 2020 with a positive SARS-CoV-2 reverse transcription polymerase chain reaction (RT-PCR) test, or with COVID-19 specific radiologic findings in case of uncertain RT-PCR status, were eligible for this retrospective study (Supplementary Figure 1). Patients that tested negative for SARS-CoV-2 in the RT-PCR test and received a different diagnosis were used as a control group. The institutional medical ethics committee provided a waiver for medical ethical law review (protocol number 20-284/C). The use of patient data for research purposes was accompanied by an opt-out procedure. All procedures performed in this study were in accordance with the 1964 Helsinki declaration and its later amendments. Blood samples from these patients were collected as a part of routine laboratory testing and platelet reactivity testing was performed within 5 h after collection. In case multiple samples were collected from one patient, the first sample after patient hospitalization was included for analysis.

### Platelet Reactivity Testing

Platelet reactivity testing was performed by diluting 5 μL whole blood (collected into heparin tubes) 1:11 dilution in 4-(2-hydroxyethyl)-1-piperazineethanesulfonic acid (HEPES) buffered saline (HBS; 10 mM HEPES, 150 mM NaCl, 1 mM MgSO4, 5 mM KCl, pH 7.4), containing either a concentration series of adenosine diphosphate ADP (0–114 μM) or PAR1-activating peptide or TRAP-6 (0–568 μM) and APC-conjugated or PE-conjugated Anti-Human P-selectin antibody clone AK4 (5 μg/mL final concentration) to detect platelet P-selectin expression, and FITC-conjugated Anti-Human fibrinogen antibody (25 μg/mL final concentration) to detect fibrinogen binding (reflects GPIIb/IIIa activation) for 30 min at room temperature. Platelet activation was stopped by fixing the sample for 20 min by 11-fold dilution into fixative (0.4% PFA in 0.9% NaCl). Samples were analyzed on a FACSCanto II using FACSDiva software version 8.0.1. Platelets were gated based on their forward and sideward scatter. We validated this gating strategy in a subset of our study cohort (12 patients) with a monoclonal antibody against the platelet-specific marker GP1bα. In this gate, 88.4% ± 6.5% (mean ± SD) was positive (defined as a fluorescence intensity >10^3^). The median fluorescence intensity (MFI) of 5,000 gated platelets was determined (Supplementary Figure 2 illustrates the gating strategy).

### Data Collection

Patient data was collected retrospectively from the day of admission to the University Medical Center Utrecht until the day of discharge or death.

### Study Variables

Demographic and clinical variables included age, sex, body mass index (BMI), comorbidities (diabetes mellitus, hypertension, hypercholesterolemia, atrial fibrillation, asthma, kidney disease and thrombotic history), immunocompromising disease and/or medication, preexisting thromboprophylaxis [direct oral anticoagulant (DOAC), vitamin K antagonist (VKA), and antiplatelet agents (ADP receptor antagonists, irreversible cyclooxygenase inhibitors, and GPIIb/IIIa inhibitors)], hospital thromboprophylaxis (all thromboprophylaxis given during hospitalization as part of standard hospital protocol), therapeutic antithrombotic medication (all agents given to treat TE) and on admission platelet count (platelet count determined within 72 h after hospitalization).

### Clinical Outcomes

Critical disease was defined according to the World Health Organization COVID-19 subgroup definitions as having clinical signs of severe pneumonia and meeting the ARDS criteria at any point during admission (13). TE diagnosed by the treating physician according to hospital protocols was a composite of acute myocardial infarction (MI), deep vein thrombosis (DVT), pulmonary thrombosis [comprises any (embolic) thrombus in the pulmonary vasculature that was diagnosed by computed tomography angiography], ischemic cerebrovascular accident (iCVA), thrombus in continuous venovenous hemofiltration (CVVH) filter, or a thrombus occurring elsewhere in the body. Patient intensive care unit (ICU) admission, mechanical ventilation, and all cause in hospital mortality was recorded for all patients.

### Statistical Analysis

Continuous variables are shown as median and interquartile range (IQR) and are compared using Mann-Whitney test. Categorical variables are shown as frequencies and percentages and are compared using Chi^2^ test (*n* < 60) or Fisher's exact test (in case of less than five samples per cell). Statistical testing was performed in IBM SPSS statistics (version 25.0.0.2).

Platelet reactivity stratified by clinical outcome was analyzed in PRISM GraphPad (version 8.3.0) by One-way ANOVA and Sidak's multiple comparisons test comparing the two groups at all concentrations of used platelet agonist.

Univariable and multivariable logistic regression models were made in IBM SPSS statistics to estimate the (adjusted) odds ratio of the clinical outcomes (TE and all-cause mortality). Most of the variables that do not associate with clinical outcome in the univariable logistic regression model were excluded from analysis in the multivariable logistic regression model. Furthermore, a variable was excluded from multivariate logistic regression if the percentage of exposure was too low or too high in order to calculate the odds ratio. Four covariates were included in the multivariable logistic regression model to avoid overfitting in the model. For the clinical outcome TE covariates were age, asthma, any preexisting thromboprophylaxis and platelet reactivity in response to a maximum concentration of TRAP-6. For the clinical outcome all-cause mortality the covariates comprised age, hypertension, any preexisting thromboprophylaxis and baseline platelet reactivity. For all tests statistical significance was defined as a two-tailed *p*- value < 0.05.

## Results

We investigated whether increased platelet reactivity is a risk factor for thrombotic events or all-cause mortality in hospitalized COVID-19 patients. We enrolled 79 SARS-CoV-2 positive patients for this study and included 21 hospitalized SARS-CoV-2 negative patients (with a different diagnosis) as a control group (Supplementary Figure 1). Platelet reactivity was assessed using blood samples collected from these patients as part of routine diagnostic testing throughout the time-course of hospitalization. We measured the expression of platelet activation marker P-selectin and platelet fibrinogen binding in response to agonist ADP and TRAP-6 (acting on the P2Y12 and PAR-1 receptor respectively).

The median age of these patients was 67 years (IQR: 54–75), more patients were male than female (57 vs. 43%, respectively) and hypertension (37%) was the most common comorbidity (Table 1). The SARS-CoV-2 negative control group had a similar median age of 67 years (IQR: 42–77) and 33% of patients were female.

**Table 1:** Patient demographics, clinical characteristics and laboratory findings.

	**All Patients (*n* = 79)**	**No TE (*n* = 57)**	**TE (*n* = 22)**	***p*-Value**	**Survivor (*n* = 66)**	**Non-survivor (*n* = 13)**	***p*-Value**
**Demographics and clinical characteristics**							
Age (years, median, IQR)	67 (54–75)	70 (59–76)	55 (46–62)	** <0.001**	65 (53–73)	75 (63–79)	**0.042**
Female (*n*, %)	34 (43)	24 (42)	10 (45)	0.788	30 (45)	4 (31)	0.33
Body mass index (kg/m^2^, median, IQR)	28 (24–31)	28 (25–32)	25 (23–29)	**0.028**	28 (24–31)	28 (26–31)	0.35
Diabetes (*n*, %)	18 (23)	15 (26)	3 (14)	0.23	14 (21)	4 (31)	0.48
Hypertension (*n*, %)	29 (37)	24 (42)	5 (23)	0.11	20 (30)	9 (69)	**0.012**
Hypercholesterolemia (*n*, %)	3 (4)	3 (5)	0 (0)	0.56	3 (5)	0 (0)	1.0
Atrial fibrillation (*n*, %)	10 (13)	10 (18)	0 (0)	0.05	7 (11)	3 (23)	0.36
Asthma (*n*, %)	10 (13)	5 (9)	5 (23)	0.13	8 (12)	2 (15)	0.67
Kidney disease (*n*, %)	8 (10)	5 (9)	3 (14)	0.68	5 (8)	3 (23)	0.12
Immunocompromised (*n*, %)	17 (22)	14 (25)	3 (14)	0.37	13 (20)	4 (31)	0.46
Thrombotic history (*n*, %)	7 (9)	6 (11)	1 (5)	0.67	6 (9)	1 (8)	1.00
Preexisting thromboprophylaxis (any, *n*, %)	35 (44)	31 (54)	4 (18)	**0.004**	26 (39)	9 (69)	**0.048**
Anticoagulant (*n*, %)	16 (20)	16 (28)	0 (0)	**0.004**	12 (18)	4 (31)	0.45
DOAC (*n*, %)	9 (11)	9 (16)	0 (0)	0.06	6 (9)	3 (23)	0.16
VKA (*n*, %)	7 (9)	7 (12)	0 (0)	0.18	6 (9)	1 (8)	1.0
Antiplatelet (*n*, %)	19 (24)	15 (26)	4 (18)	0.49	14 (21)	5 (38)	0.28
Acetylsalicylic acid (*n*, %)	12 (15)	10 (18)	2 (9)	0.49	8 (12)	4 (31)	0.10
Clopidogrel (*n*, %)	9 (11)	6 (11)	3 (14)	0.70	7 (11)	2 (15)	0.64
Preexisting medication influencing platelets (*n*, %)	10 (13)	9 (16)	1 (5)	0.27	7 (11)	3 (23)	0.36
Disease severity (critical, *n*, %)	67 (85)	48 (84)	19 (86)	1.0	54 (82)	13 (100)	n.a.
**Laboratory findings**							
Platelet count on hospital admission (G/L, median, IQR)	221 (150–298) *n* = 57	208 (137–298) *n* = 43	250 (158–384) *n* = 14	0.321	221 (151–290) *n* = 46	221 (134–303) *n* = 11	0.97

*DOAC, direct oral anticoagulant; IQR, interquartile range; VKA, vitamin K antagonist; TE, thrombotic event. Bold p-values indicate a significant p-value (p < 0.05)*.

Most patients (85%) developed severe COVID-19 disease according to the World Health Organization guideline (13). Half of the patients (51%) were admitted to the ICU and 44% of all patients required mechanical ventilation. Almost all patients (96%) received hospital thromboprophylaxis. However, 28% of the patients developed one or more TE, which in most cases was a pulmonary thrombosis (Table 2). In this study, TE predominantly (69%) occurred during ICU stay (Supplemental Table 1).

**Table 2:** Patient treatment and clinical outcomes.

	**All Patients (*n* = 79)**	**No TE (*n* = 57)**	**TE (*n* = 22)**	***p*-Value**	**Survivor (*n* = 66)**	**Non-survivor (*n* = 13)**	***p*-Value**
**Treatment**							
Hospital prophylaxis (any, *n*, %)	76 (96)	56 (98)	20 (91)	0.19	63 (95)	13 (100)	1.0
LMWH (*n*, %)	69 (87)	50 (88)	19 (86)	1.0	57 (86)	12 (92)	1.0
VKA (*n*, %)	5 (6)	5 (9)	0 (0)	0.31	4 (6)	1 (8)	1.0
DOAC (*n*, %)	9 (11)	9 (16)	0 (0)	0.56	6 (9)	3 (23)	0.16
Therapeutic heparin (*n*, %)	11 (14)	–	11 (50)	–	9 (14)	2 (15)	1.0
Therapeutic LMWH (*n*, %)	19 (24)	–	19 (86)	–	18 (27)	1 (8)	0.17
Therapeutic DOAC (*n*, %)	17 (22)	–	17 (77)	–	17 (26)	0 (0)	0.06
Therapeutic VKA (*n*, %)	1 (1)	–	1 (5)	–	1 (2)	0 (0)	1.0
Thrombolytic agent (*n*, %)	1 (1)	–	1 (5)	–	1 (2)	0 (0)	1.0
**Clinical Outcome**							
TE (any, *n*, %)	22 (28)	–	22 (100)	–	20 (30)	2 (15)	0.33
One (*n*, %)	18 (23)	–	18 (82)	–	16 (24)	2 (15)	0.72
Two (*n*, %)	4 (5)	–	4 (18)	–	4 (6)	0 (0)	1.0
DVT (*n*, %)	4 (5)	–	4 (18)	–	4 (6)	0 (0)	1.0
Pulmonary thrombosis (*n*, %)	15 (19)	–	15 (68)	–	15 (23)	0 (0)	0.06
MI (*n*, %)	0 (0)	–	0 (0)	–	0 (0)	0 (0)	–
iCVA (*n*, %)	2 (3)	–	2 (9)	–	2 (3)	0 (0)	1.0
Thrombus in other location (*n*, %)	3 (4)	–	3 (14)	–	3 (5)	0 (0)	1.0
Thrombus in CVVH filter (*n*, %)	2 (3)	–	2 (9)	–	0 (0)	2 (15)	**0.025**
Indication for ICU (*n*, %)	50 (63)	32 (56)	18 (82)	**0.034**	37 (56)	13 (100)	**0.002**
ICU admission (*n*, %)	40 (51)	22 (39)	18 (82)	** <0.001**	37 (56)	3 (23)	**0.030**
ICU length of stay (days, median, IQR)	12 (8–19)	9 (5–16)	16 (12–23)	**0.007**	12 (8–17)	22(20–33)	**0.031**
Mechanical ventilation	35 (44)	18 (32)	17 (77)	** <0.001**	32 (48)	3 (23)	0.09
Duration of mechanical ventilation (days, median, IQR)	11 (7–17)	9 (6–14)	14 (10–21)	**0.028**	11 (7–15)	20(6–33)	0.35
Hospital length of stay (days, median, IQR)	17(10–26)	13(8–21)	27(20–38)	** <0.001**	17(12–28)	13(8–24)	0.23
Mortality (*n*, %)	13 (16)	11 (19)	2 (9)	0.33	–	–	–

*CVVH, continuous venovenous hemofiltration; DOAC, direct oral anticoagulant; DVT, deep vein thrombosis; IQR, interquartile range; LMWH, low molecular weight heparin; MI, myocardial infraction; ICU, intensive care unit; iCVA, ischemic cerebrovascular accident; VKA, vitamin K antagonist; TE, thrombotic event. Bold p-values indicate a significant p-value (p < 0.05)*.

Remarkably, patients that developed a TE were younger [median age, 55 (IQR: 46–62) vs. 70 (IQR: 59–76) years; *p* = < 0.001] and had a lower body mass index [median, 25 (IQR: 23–29) vs. 28 (IQR: 25–32); *p* = 0.028; Table 1], than patients that did not develop a TE. From this perspective, it is noteworthy that patients that developed at TE used less preexisting thromboprophylaxis prior to hospitalization and unrelated to SARS-CoV-2 infection (18 vs. 54%; *p* = 0.004; Table 1).

All-cause mortality in this study was 16% (Table 2). Non-survivors were older [median age, 75 (IQR: 63–79) vs. 65 (IQR: 53–73) years; *p* = 0.042; Table 1] and more often had hypertension (69% vs. 30% *p* = 0.012; Table 1).

**Figure 1:**
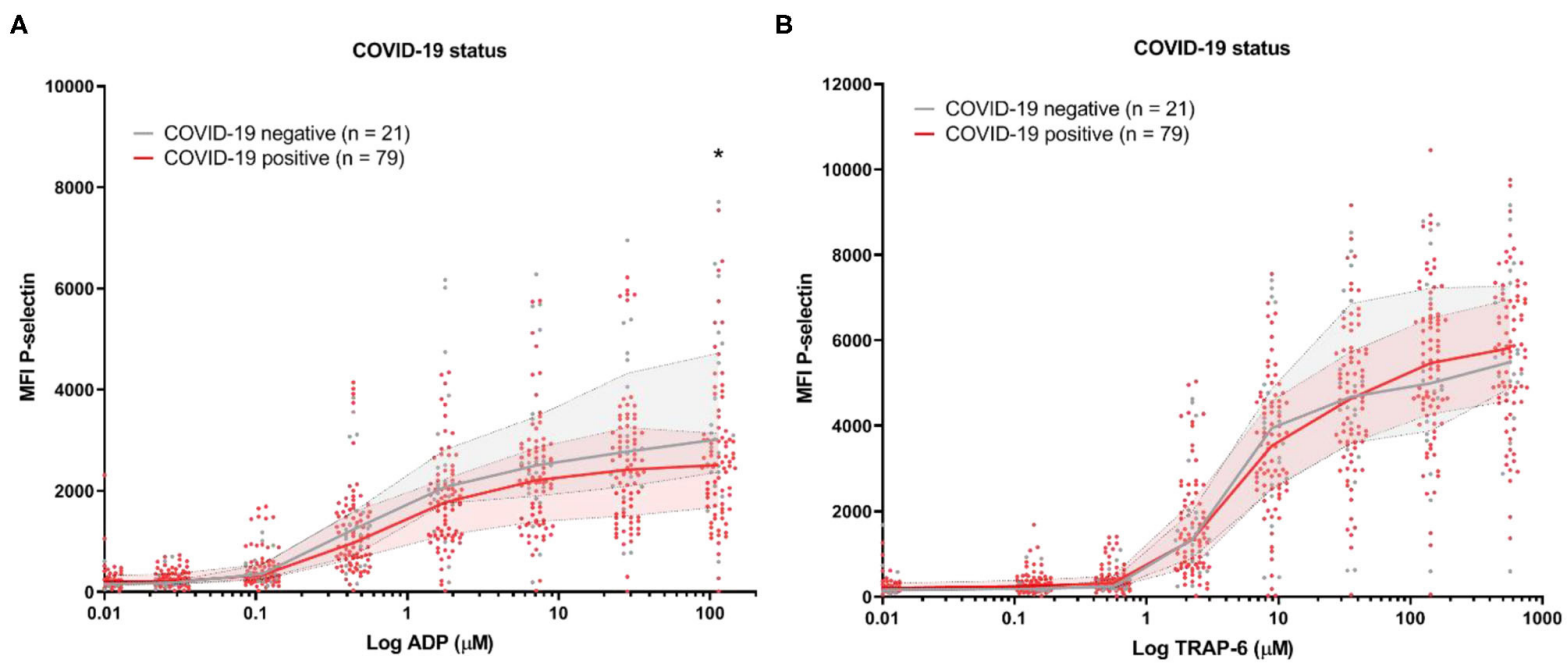
Platelet reactivity of hospitalized COVID-19 positive patients and COVID-19 negative control patients with a different diagnosis. Median fluorescence intensity (MFI) of platelet P-selectin expression in response to ADP **(A)**, or TRAP-6 **(B)**. Data points represent individual values of COVID-19 positive patients (*n* = 79; displayed in red) or COVID-19 negative control patients (*n* = 21; displayed in gray). The median value and interquartile range are indicated. Statistical difference was tested for all concentrations of agonist using one-way ANOVA with Sidak's multiple comparisons test. *p < 0.05.

Platelet reactivity assessed by P-selectin expression in response to ADP and TRAP-6 was similar for COVID-19 positive patients compared to the COVID-19 negative control patients (Figure 1). In contrast, when we investigated platelet reactivity through fibrinogen binding, we found this was decreased in COVID-19 patients (Supplemental Figure 3). This is in line with earlier studies (9, 14). However, our analytical method is sensitive to increased plasma fibrinogen levels (Supplementary Figure 4), which is commonly seen in COVID-19 and might affect our outcomes (5). We therefore continued to exclusively investigate platelet reactivity by triggered P-selectin expression.

**Figure 2:**
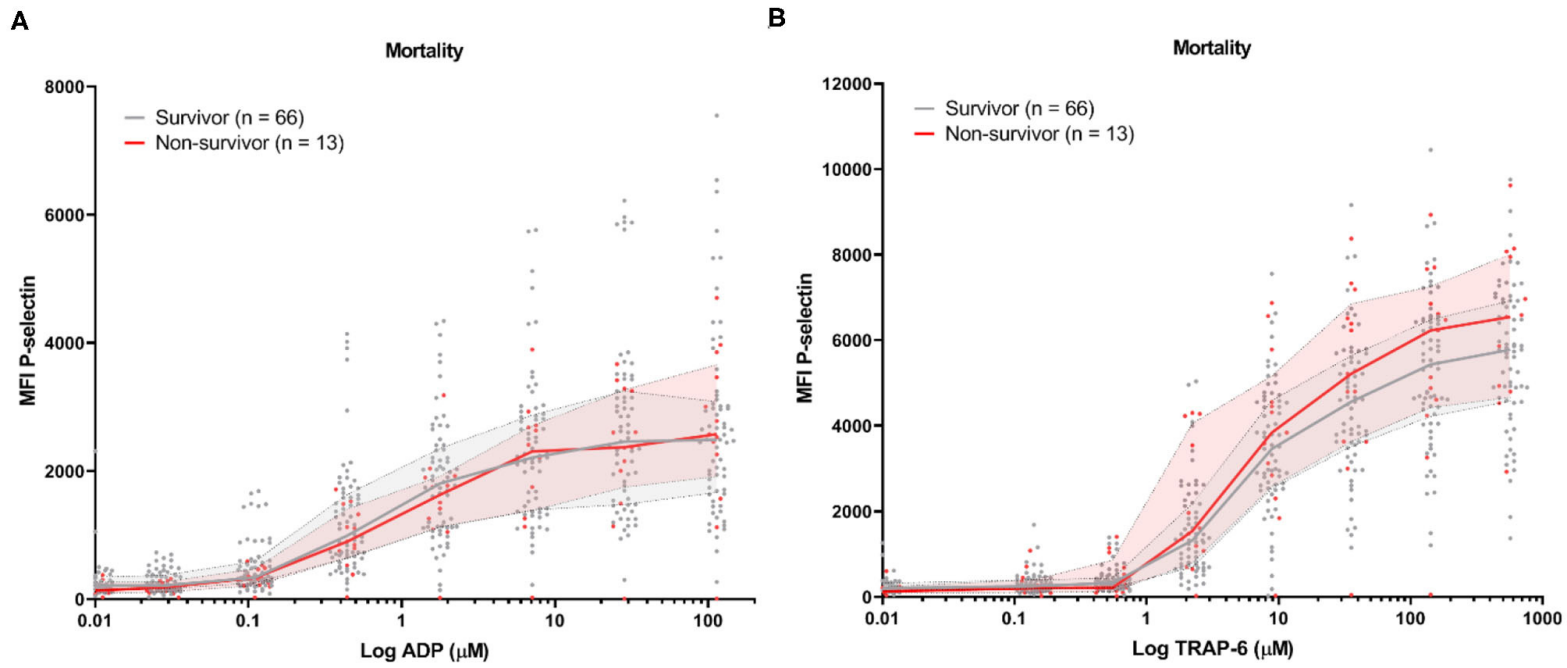
COVID-19 patient platelet reactivity stratified by all-cause mortality. Median fluorescence intensity (MFI) of platelet P-selectin expression in response to ADP **(A)**, or TRAP-6 **(B)**. Data points represent individual values of non-survivors (*n* = 13; displayed in red) or survivors (*n* = 66; displayed in gray). The median value and interquartile range are indicated. Statistical difference was tested for all concentrations of agonist using one-way ANOVA with Sidak's multiple comparisons test. No significant differences were found.

**Figure 3:**
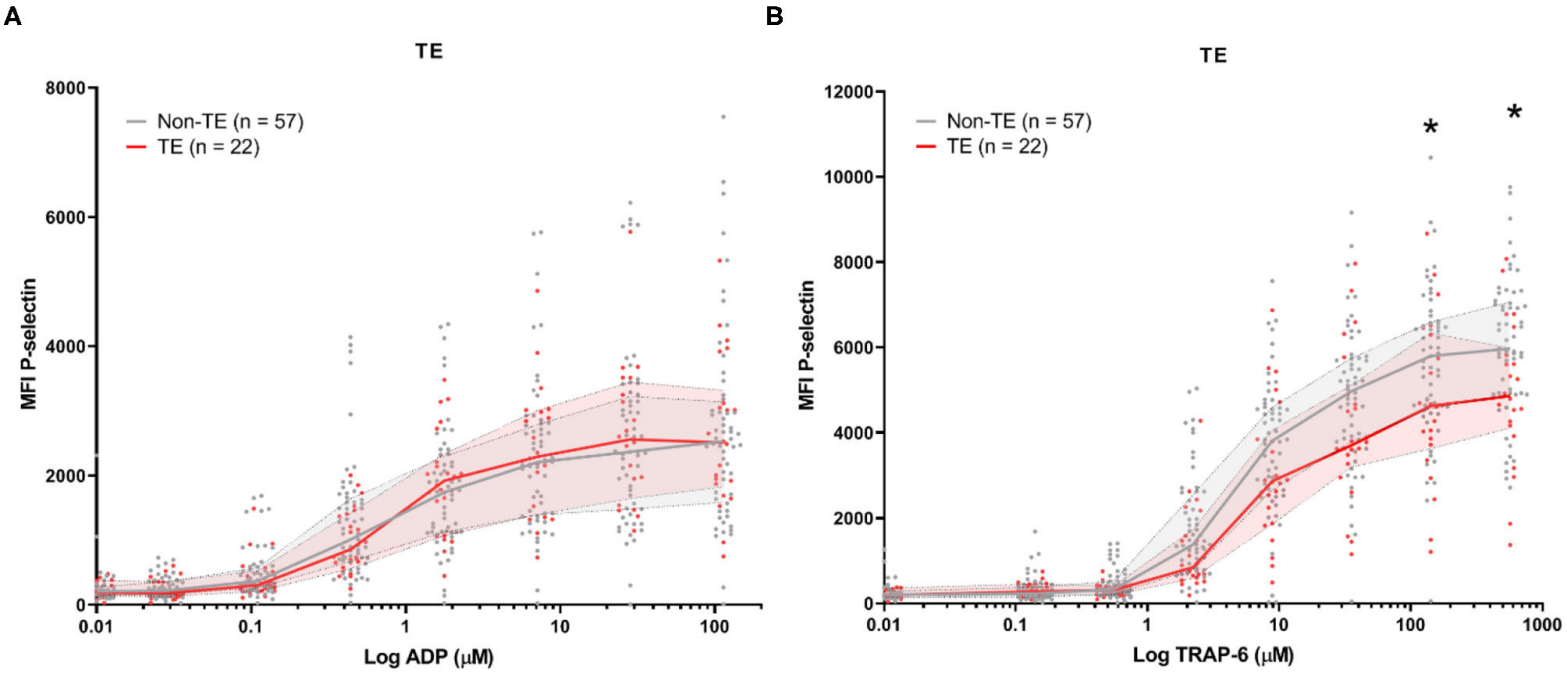
COVID-19 patient platelet reactivity stratified by thrombotic events (TE). Median fluorescence intensity (MFI) of platelet P-selectin expression in response to ADP **(A)**, or TRAP-6 **(B)**. Data points represent individual values of patients that do not develop TE (*n* = 57; displayed in gray) or do develop TE during hospitalization (*n* = 22; displayed in red). The median value and interquartile range are indicated. Statistical difference was tested for all concentrations of agonist using one-way ANOVA with Sidak's multiple comparisons test. *p < 0.05.

Baseline platelet P-selectin expression was similar for patients stratified by all-cause mortality (Figure 2) or by the development of TE (Figure 3). P–selectin expression in response to ADP and TRAP-6 was similar for patients stratified by all-cause mortality (Figure 2). However, when patients were stratified by TE development, we observed that platelet reactivity triggered by high concentrations of TRAP-6 was decreased in patients that developed a TE (Figure 3B).

Univariable logistic regression analysis revealed that the risk for developing TE was decreased in patients taking preexisting thromboprophylaxis [odds ratio (OR) = 0.19, 95% CI 0.06–0.62; *p* = 0.006], with higher age (OR = 0.94, 95% CI 0.91–0.98; *p* = 0.003; per 1 year increase) and with higher platelet reactivity in response to TRAP-6 (OR = 0.97, 95% CI 0.94–1.00; *p* = 0.032, per 100 unit MFI increase; Table 3). In good correspondence to earlier studies (15), the risk of all-cause mortality was higher in patients with hypertension (OR = 5.18, 95% CI 1.43–18.8; *p* = 0.012).

**Table 3:** Risk factors associated with TE and all-cause mortality.

	**Clinical outcome TE**				**Clinical outcome all-cause mortality**			
	**Univariable OR (95% CI)**	***p*-Value**	**Multivariable AOR (95% CI)**	***p*-Value**	**Univariable OR (95% CI)**	***p*-Value**	**Multivariable AOR (95% CI)**	***p*-Value**
**Demographics and clinical characteristics**								
Age, years	**0.94 (0.91–0.98)**	**0.003**	**0.96 (0.92–1.00)**	**0.041**	1.05 (1.00–1.10)	0.079	1.03 (0.97–1.10)	0.328
Female (vs. male)	1.15 (0.43–3.08)	0.788	–	–	0.53 (0.15–1.91)	0.533	–	–
Body mass index	0.94 (0.85–1.04)	0.209	–	–	1.03 (0.93–1.13)	0.600	–	–
Diabetes	0.44 (0.11–1.71)	0.237	–	–	1.66 (0.44–6.16)	0.456	–	–
Hypertension	0.40 (0.13–1.25)	0.115	–	–	**5.18 (1.43–18.8)**	**0.012**	3.04 (0.78–11.9)	0.109
Hypercholesterolemia	–^**^	–	–	–	–		–	–
Atrial fibrillation	–^**^	–	–	–	2.53 (0.56–11.4)	0.228	–	–
Asthma	3.06 (0.79–11.7)	0.106	**6.2 (1.15–33.7)**	**0.034**	1.32 (0.25–7.01)	0.747	–	–
Kidney disease	1.64 (0.36–7.54)	0.524	–	–	3.66 (0.75–17.8)	0.107	–	–
Immunocompromised	0.48 (0.12–1.89)	0.297	–	–	1.81 (0.48–6.82)	0.379	–	–
Thrombotic history	0.40 (0.05–3.57)	0.416	–	–	2.01 (0.09–7.57)	0.833	–	–
Preexisting thromboprophylaxis present (vs not present)	**0.19 (0.06–0.62)**	**0.006**	**0.19 (0.04–0.84)**	**0.029**	3.46 (0.97–12.4)	0.057	1.70 (0.37–7.8)	0.490
Anticoagulant	–^**^	–	–	–	2.00 (0.53–7.59)	0.308	–	–
DOAC	–^**^	–	–	–	3.00 (0.64–14.0)	0.162	–	–
VKA	–^**^	–	–	–	0.83 (0.09–7.57)	0.871	–	–
Antiplatelet	0.62 (0.18–2.14)	0.451	–	–	2.32 (0.66–8.21)	0.191	–	–
Acetylsalicylic acid	0.47 (0.09–2.34)	0.357	–	–	3.22 (0.80–12.9)	0.099	–	–
Clopidogrel	1.34 (0.30–5.91)	0.697	–	–	1.53 (0.28–8.37)	0.622	–	–
Preexisting medication influencing platelets present (vs. not present)	0.25 (0.03–2.13)	0.207	–	–	2.53 (0.56–11.4)	0.228	–	–
**Laboratory findings**								
Platelet count on hospital admission (G/L)	1.00 (1.00–1.01)	0.275	–	–	1.00 (0.99–1.00)	0.770	–	–
Platelet reactivity baseline^*^	0.96 (0.75–1.22)	0.737	–	–	0.54 (0.28–1.04)	0.066	0.61 (0.32–1.20)	0.150
Platelet reactivity in response to TRAP-6^*^	**0.97 (0.94–1.00)**	**0.032**	**0.96 (0.93–0.99)**	**0.020**	1.00 (0.98–1.04)	0.600	–	–
Platelet reactivity in response to ADP^*^	1.00 (0.96–1.03)	0.835	–	–	1.00 (0.96–1.04)	0.890	–	–

*DOAC, direct oral anticoagulant; IQR, interquartile range; MFI, median fluorescence intensity; VKA, vitamin K antagonist; TE, thrombotic event*.

* *Per 100 unit increase*.

** *n.a. due to perfect separation. Bold p-values indicate a significant p-value (p < 0.05)*.

After covariate adjustment for age, the risk for TE remained decreased in patients taking preexisting thromboprophylaxis (AOR = 0.19, 95% CI 0.04–0.84; *p* = 0.029; Table 3). Furthermore, patients with asthma have a strong risk on developing TE (AOR = 6.2, 95% CI 1.15–33.7; *p* = 0.034; Table 3), without impacting all-cause mortality.

**Figure 4:**
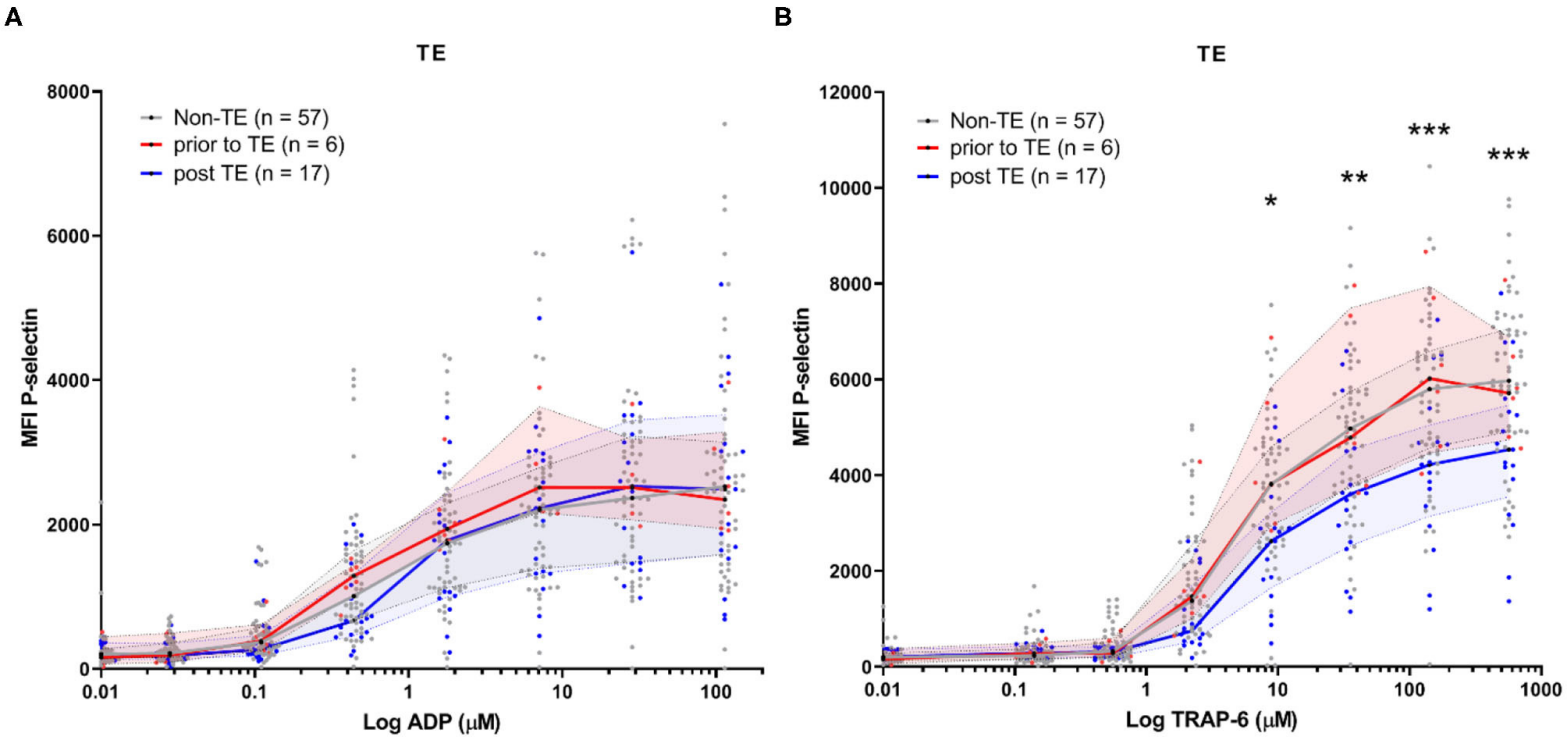
COVID-19 patient platelet reactivity of patients that develop (a) thrombotic event(s) (TE) stratified prior or after the occurrence of the TE. Median fluorescence intensity (MFI) of platelet P-selectin expression in response to ADP **(A)**, or TRAP-6 **(B)**. Data points represent individual values of platelet reactivity of patients prior to the occurrence of a TE (*n* = 6; displayed in red), or after the TE (*n* = 17; displayed in blue). For one patient platelet reactivity was measured both prior to and after experiencing TE. Both datapoints are included. For reference, platelet reactivity of patients that do not develop a TE (*n* = 57) is displayed in gray. The median value and interquartile range are indicated. Statistical difference was tested using one-way ANOVA with preselected multiple comparisons (comparing platelet reactivity of post TE to non-TE) using Sidak's multiple comparisons test. *p < 0.05; **p < 0.005; ***p < 0.0005.

In our study, we assessed platelet reactivity in study samples at variable time-points throughout hospitalization (samples were collected as part of routine diagnostics).This means that patient samples could be collected either before or after TE development. We therefore stratified platelet reactivity results by the moment of sample collection; either before or after TE development (Figure 4). We found platelet reactivity in response to ADP was similar (Figure 4A). In contrast, platelet reactivity in response to TRAP-6 was lowered in samples analyzed after TE occurrence (Figure 4B). As a result, a lower platelet reactivity in response to TRAP-6 is associated with an increased risk for developing TE (AOR = 0.96, 95% CI 0.93–0.99; *p* = 0.020; per 100 unit MFI increase; Table 3).

## Discussion

We studied platelet reactivity in a retrospective cohort study of 79 hospitalized COVID-19 patients. This study was limited by the fact that we were unable to include healthy controls during the early stages of the pandemic for comparison. As an alternative, we included hospitalized patients that tested negative for COVID-19 and received a different diagnosis. This hospitalized control group has a similar age and sex distribution compared to our COVID-19 patients [68 years (IQR: 42–76), 33% female]. However, information on the development of TE in the control group was unavailable for this study. No difference in platelet reactivity was seen between the COVID-19 positive and negative control group of hospitalized patients based on P-selectin expression (Figure 1). By comparison, other studies showed that the fraction of platelets that are positive for P-selectin is elevated in COVID-19 patients (~4%) compared to healthy controls (~2%) (9).

Similarly, we found that both baseline and triggered P-selectin expression by platelets were similar between survivors and non-survivors in this study (Figure 2) and our multivariate analysis failed to identify (triggered) P-selectin expression as a risk factor for all-cause mortality (Table 3).

We observed that platelet fibrinogen binding (detected with a fluorescently labeled polyclonal antibody) was decreased in COVID-19 patients compared to COVID-19 negative control patients, which suggested a decreased activation of GPIIb/IIIa (Supplementary Figure 3). Similar results were previously reported using PAC-1 antibody (9, 14). However, strongly elevated plasma fibrinogen levels have been reported in COVID-19 patients (5). As we identified that our assay method is sensitive to variation in fibrinogen levels (Supplementary Figure 4), we cannot report on the activation of GPIIb/IIIa in our patient cohort.

The incidence of TE (28%) and its predominant occurrence during ICU stay in this study is in line with other studies (7, 16). Only 2 out of 15 patients with pulmonary thrombosis that were found in this study, had an accompanying confirmed DVT (13%). However, it cannot be ruled out that some of the remaining patients experienced non-symptomatic DVT. An autopsy study of 11 deceased COVID-19 patients without clinically suspected VTE identified small and mid-sized pulmonary artery thrombosis (17). This suggests that TE can occur *in situ* and is in line with findings from other studies (7, 8). Severe arterial thrombotic events have been reported in patients with COVID-19 (18). Two of the patients in this study experienced an iCVA, while one patient developed a thrombus in the aortic arch accompanied by ischemia of the lower extremities.

Indes et al. compared arterial thrombosis occurring in COVID-19 positive and negative patients and found that COVID-19 positive patients had higher D-dimer levels, were younger (64 vs. 70 years of age) and less often used preexisting antiplatelet medication compared to the negative control group (21 vs. 63%) (19). In a similar manner, we found that COVID-19 patients taking preexisting thromboprophylaxis had a lower risk for TE development (AOR = 0.19, 95% CI 0.04–0.84 *p* = 0.029; Table 3).

The protective effect of preexisting thromboprophylaxis is limited to TE development: a recent study by Tremblay et al. found that anticoagulation prior to COVID-19 infection did not influence the risk on all-cause mortality (20). Our study confirms this finding: pre-existing thromboprophylaxis does not improve mortality after adjustment for age and other confounding risk factors (Table 3). It should be noted that the patient population studied by Tremblay et al. was different from ours. It had a lower rate of thrombosis (1.2%) and mortality (15%). Where our study exclusively contained hospitalized patients that were mostly (96%) treated with in-hospital anticoagulation, only 54% of the COVID-19 patients in the study of Tremblay et al. were admitted to the hospital. This subset of hospitalized patients may have received in-hospital anticoagulation while the non-hospitalized patients did not. It is also possible that the low rate of thrombosis seen in their study population is the result of a relatively mild disease severity. This confirms other studies indicating the relationship between COVID-19 disease severity and risk on developing thrombosis and suggest that any protective impact of prior thromboprophylaxis is mainly visible in a patient population that is at risk for developing thrombosis (7).

In this study, we found that platelet reactivity is mainly lowered *after* TE development (Figure 4B). This observation is specific to platelet activation by TRAP-6. TRAP-6 engages PAR-1 and activates it. Normally, this receptor is cleaved by thrombin, resulting in the formation of a tethered ligand for activation (21). Exposure of platelets to thrombin desensitizes them, rendering them insensitive to TRAP-6 (22). The decreased platelet P-selectin expression in response to TRAP-6 (>10 μM), seen in patients after developing a TE in comparison to patients that did not develop a TE might be explained due to platelet desensitization *in vivo* as a result of thrombin exposure.

However, other enzymes can also induce PAR-1 desensitization. These include plasmin and neutrophil elastase (which can be released during NET formation), which can cleave and truncate the N-terminus of PAR-1 including the thrombin cleavage site, rendering PAR-1 unable to be activated by thrombin (23, 24). Both elevated plasmin-α2-antiplasmin complex levels and evidence for neutrophil degranulation and NETs formation have been reported in COVID-19 patient plasma and tissue (25, 26). This suggests that PAR-1 desensitization may take place secondary to COVID-19 associated thromboinflammatory events.

In conclusion, our study revealed that platelet reactivity in COVID-19 patients was lowered after TE development, possibly as a result of *in vivo* desensitization by thrombin. Patients taking any preexisting thromboprophylaxis prior to hospitalization had a 5-fold lower risk for TE development (AOR = 0.19, 95% CI 0.04–0.84; *p* = 0.029). This suggests that this might be beneficial for coping with COVID-19 associated hypercoagulability, and highlights the importance of therapy adherence in the general population.

## Data Availability Statement

The raw data supporting the conclusions of this article will be made available by the authors, without undue reservation.

## Ethics Statement

The studies involving human participants were reviewed and approved by Medisch Ethische Toetsingscommissie (METC) Utrecht. Written informed consent for participation was not required for this study in accordance with the national legislation and the institutional requirements.

## Author Contributions

CC, AB, SSm, CS, SV, NP, SSe, AK, and AW designed and performed the experiments. BJ, JS, NJ, HG, RS, and SB gathered samples and clinical data. CC and BJ analyzed the data and wrote, with support from all other authors, the manuscript. SM, SN, MB, EV, LK, KK, and CM designed the study and supervised the project. All authors contributed to the article and approved the submitted version.

## Dutch Covid & Thrombosis Coalition Contributors

### Amsterdam Universitaire Medische Centra

-AMC: Dr. M. Coppens; Prof. Dr. N. P. Juffermans; Prof. Dr. S. Middeldorp

-VUMC: Prof. Dr. C. M. P. M. Hertogh; J. G. Hugtenburg; Dr. E. J. Nossent

### Erasmus Medisch Centrum

Drs. J. van den Akker; Dr. R. Bierings; Dr. H. Endeman; Dr. M. Goeijenbier; Prof. Dr. D. A. M. P. J. Gommers; Prof. Dr. M. P. G. Koopmans; Prof. Dr. T. Kuiken; T. Langerak, PhD candidate; Dr. M. N. Lauw; Prof. Dr. M. P. M. de Maat; D. Noack, PhD candidate; M. P. Raadsen, PhD candidate; Dr. B. Rockx; C. Rokx; K. Tong-Minh, PhD candidate; Dr. L van der Toorn; C. A. den Uil

### Farmadam

Tineke Roest, coordinating pharmacist.

### Institute of Research of Hospital de la Santa Creu i Sant Pau, Barcelona

Dr. J. Manuel Soria, Unit of Genomics of Complex Diseases.

### Leids Universitair Medisch Centrum

M. L. Antoni; Dr. M. Bos; Drs. Burggraaf, PhD candidate; Prof. S. C. Cannegieter; Prof. Dr. H. C. J. Eikenboom; Dr. P. L. den Exter; Dr. J. J. M. Geelhoed; Prof. Dr. M. V. Huisman; Prof. E. de Jonge; Drs. F. H. J. Kaptein, PhD candidate; Dr. F. A. Klok; Dr. L. J. M. Kroft; Drs. L. Nab, PhD candidate; Dr. M. K. Ninaber; Prof. Dr. H. Putter; Dr. A. M. da Rocha Rondon; Dr. A. H. E. Roukens; Drs. M. A. M. Stals; PhD candidate; Prof. Dr. H. H. Versteeg; Dr. H. W. Vliegen; Dr. B. J. M. van Vlijmen.

### Maastricht Universitair Medisch Centrum

Dr. B. C. T. van Bussel; Prof. Dr. T. M. Hackeng; Drs. T. van de Berg; Prof. Dr. H. ten Cate; Dr. ir. Y. Henskens; Dr. H. Spronk; Prof. Dr. L. Schurgers; Drs. R. Bruggemann; Dr. B. Spaetgens; Dr. K. Winckers; Drs. R. Olie; Prof. Dr. M.A. Spruit.

### Radboud Universitair Medisch Centrum

Dr. J. Leentjens; Dr. Q. de Mast

### Sanquin Research, Amsterdam

Dr. M. van den Biggelaar; Prof. Dr. J. C. M. Meijers (Amsterdam Universitaire Medische Centra); Prof. Dr. J. Voorberg (Amsterdam Universitaire Medische Centra).

### Synapse Research Institute

Dr. B. de Laat.

### Trombosedienst Maastricht

Dr. A. Ten Cate-Hoek.

### Universitair Medisch Centrum Groningen

Prof. Dr. T. Lisman; Prof. Dr. K. Meijer.

### Universitair Medisch Centrum Utrecht

Prof. Dr. O. L. Cremer, Dr. G. Geersing, Prof Dr. H. A. H. Kaasjager, Dr. N. Kusadasi, Dr. A. Huisman, Dr. M. Nijkeuter, Prof. Dr. R. E. G. Schutgens, Dr. R. T. Urbanus, Dr. J. Westerink.

## COVPACH Study Group

### Department of Trauma Surgery

B. Bindels; T. M. P. Nijdam; N. L. M. van de Ven; R. Verhaegh; B. W. Verboeket; D. Laane; K.van Wessem; F. Hietbrink; L. P. H. Leenen

### Department of Respiratory Medicine

D. E. J. van Spengler; W. Buitenwerf; G. Giustarini; E. Mulder; H. Heijerman.

### Department of Internal Medicine

A. D. Zabaleta; F. van den Bos; F. Stiphout.

### Department of Intensive Care

E. Rademaker; M. R. J. Varkila; N. de Mul; O. L. Cremer; A. Slooter.

### Center for Translational Immunology

E. M. Delemarre; M. Limper; F. van Wijk; A. Pandit; H. Leavis; N. Vrisekoop.

### Central Diagnostic Laboratory

S. Haitjema; I. E. Hoefer.

## Conflict of Interest

The authors declare that the research was conducted in the absence of any commercial or financial relationships that could be construed as a potential conflict of interest.

## Abbreviations

ADP: adenosine diphosphate
AOR: adjusted odds ratio
APC: Allophycocyanin
ARDS: acute respiratory distress syndrome
BMI: body mass index
COVID-19: coronavirus disease of 2019
CVD: cardiovascular disease
CVVH: continuous venovenous hemofiltration
DOAC: direct oral anticoagulant
DVT: deep vein thrombosis
GPIIb/IIIa: glycoprotein IIb/IIIa
HBS: HEPES buffered saline
HEPES: 4-(2-hydroxyethyl)-1-piperazineethanesulfonic acid
ICU: intensive care unit
iCVA: ischemic cerebrovascular accident
IQR: interquartile range
LMWH: low molecular weight heparin
MFI: median fluorescence intensity
MI: myocardial infarction
OR: odds ratio
PAR: protease-activated receptor
RT-PCR: reverse transcription polymerase chain reaction
SARS-CoV-2: severe acute respiratory syndrome coronavirus 2
TE: thrombotic event
TRAP-6: Thrombin Receptor Activator Peptide 6.
