# Supplementary material for: Thrombotic Events in COVID-19 Are Associated With a Lower Use of Prophylactic Anticoagulation Before Hospitalization and Followed by Decreases in Platelet Reactivity
Source: Front Med (Lausanne). 2021 Apr 22;8:650129. doi: 10.3389/fmed.2021.650129 (PMC8100661; doi:10.3389/fmed.2021.650129)
Supplement: Supplementary file 1 [file Data_Sheet_1.docx]

Supplemental Material

# Supplemental Figures

Received samples from n = 133 hospitalized patients between March 17th – May 1st 2020 with suspected COVID-19 infection

Included the first sample after hospitalization that was analyzed within 5 hours from n = 21 COVID-19 negative patients who received a different diagnosis.

Included the first sample after hospitalization that was analyzed within 5 hours from n = 79 patients with confirmed COVID-19 infection.

**Supplemental Figure 1:** Study patient sample inclusions.


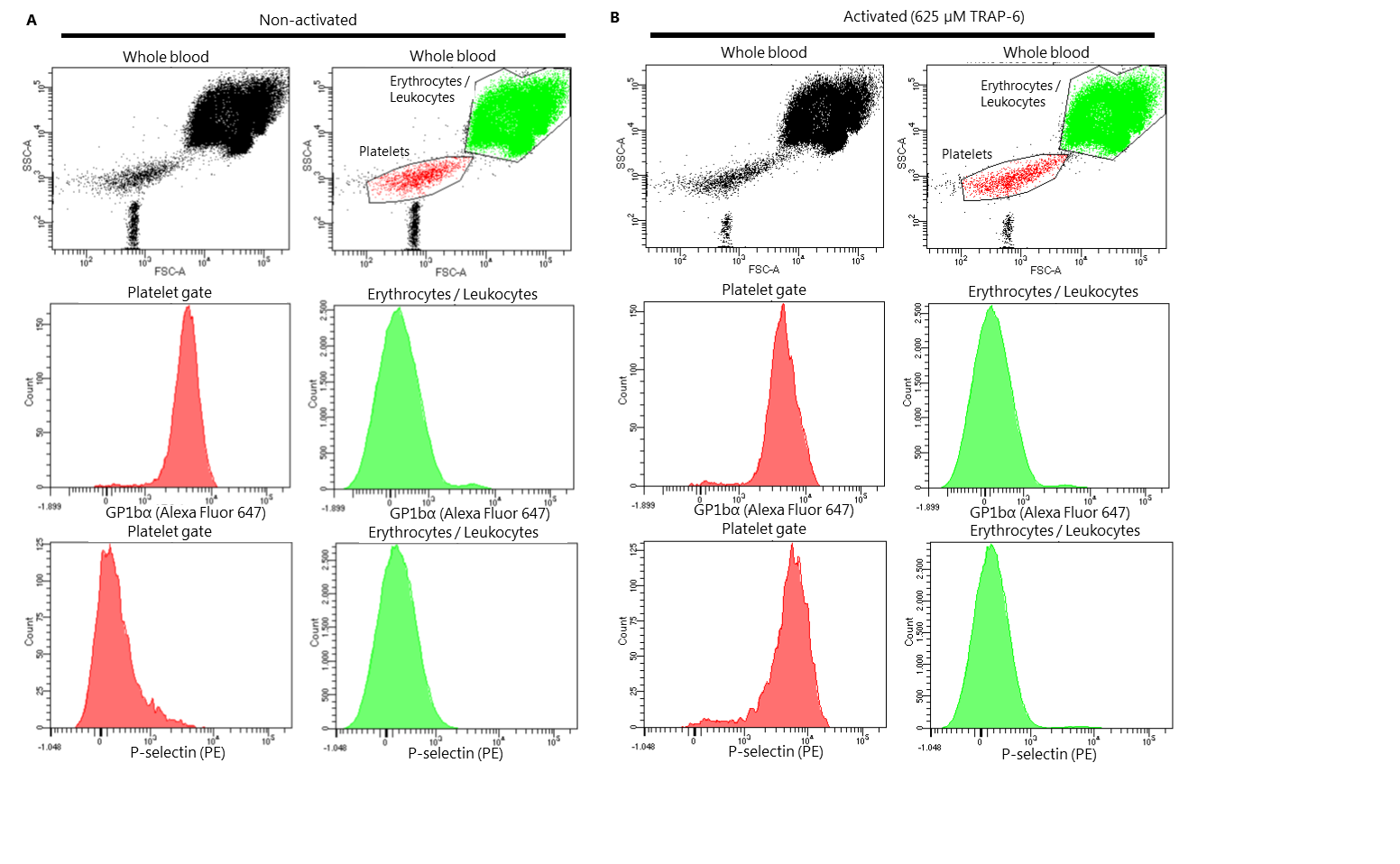


**Supplemental Figure 2:** FACS gating strategy. Platelets are gated based on their forward scatter (FSC) and sideward scatter (SSC). In a subset of samples (12/79) a platelet specific marker CD42b (Glycoprotein 1bα) was included to validate our FACS gating strategy. Cells in the platelet gate (indicated in red) are positive for platelet marker GP1bα. Non-activated platelets express low levels of activation marker P-selectin (**A**), which increases upon platelet activation (**B**). Cells in the erythrocyte and leukocyte gate are negative for the platelet marker GP1bα and P-selectin.

**
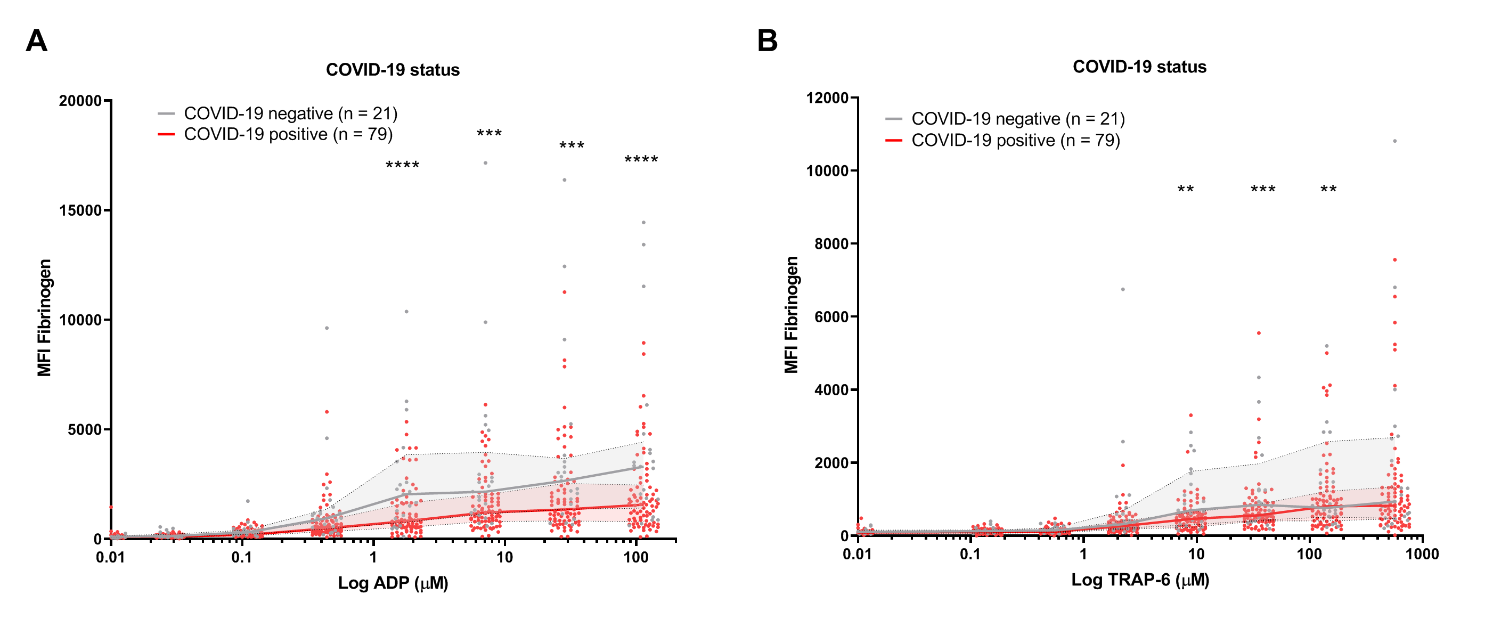
**

**Supplemental Figure 3:** Patient platelet reactivity stratified by COVID-19 status. Median fluorescence intensity (MFI) of platelet fibrinogen binding in response to ADP (**A**), or TRAP-6 (**B**). Data points represent individual values of patients that are COVID-19 negative and received a different diagnosis (n = 21; displayed in grey) or patients that are COVID-19 positive (n = 79; displayed in red). The median value and interquartile range (IQR) are indicated. Statistical difference was tested for all concentrations of agonist using one-way ANOVA with Sidak’s multiple comparisons test. ** = *p* <0.005; *** = *p* <0.0005; **** = *p* <0.0001.

**Supplemental Figure 4:** Supplementation of exogenous fibrinogen to healthy donor blood (under approval by the local medical ethics committee with written informed consent). Median fluorescence intensity (MFI) of platelet bound fibrinogen (**A,B**) in response to ADP (**A**), or TRAP-6 (**B**). Mean and standard error of healthy citrated donor blood (n = 3) supplemented with buffer control (final concentration of 3 µg/mL fibrinogen; displayed in grey), or exogenous human fibrinogen diluted in PBS buffer (Enzyme Research Laboratories, South Bend, Indiana, USA) prior to platelet function testing (final concentrations of 4,5; 9, or 15 µg/mL fibrinogen, displayed in blue, red and orange respectively).

# Supplemental Tables

| TE | Total | No ICU stay | Prior to ICU stay | During ICU stay without mechanical ventilation | During ICU stay during mechanical ventilation | During ICU stay after mechanical ventilation | After ICU stay after mechanical ventilation |
| --- | --- | --- | --- | --- | --- | --- | --- |
| DVT (n, %) | 4 | 0 (0) | 0 (0) | 0 (0) | 3 (75) | 0 (0) | 1 (25) |
| Pulmonary thrombosis (n, %) | 15 | 3 (20) | 0 (0) | 1 (7) | 8 (53) | 1 (7) | 2 (13) |
| iCVA (n, %) | 2 | 0 (0) | 0 (0) | 0 (0) | 2 (100) | 0 (0) | 0 (0) |
| Thrombus in other location (n, %) | 3 | 1 (33) | 1 (33) | 0 (0) | 1(33) | 0 (0) | 0 (0) |
| Thrombus in CVVH filter (n, %) | 2 | 0 (0) | 0 (0) | 0 (0) | 2 (100) | 0 (0) | 0 (0) |
| Abbreviations: CVVH, continuous venovenous hemofiltration; DVT, deep vein thrombosis; ICU, intensive care unit; iCVA, ischemic cerebrovascular accident; TE, thrombotic event. | | | | | | | |

**Supplemental Table 1:** Thrombotic events in relation to intensive care unit (ICU) stay.
